# Novel Polyfermentor Intestinal Model (PolyFermS) for Controlled Ecological Studies: Validation and Effect of pH

**DOI:** 10.1371/journal.pone.0077772

**Published:** 2013-10-30

**Authors:** Annina Zihler Berner, Susana Fuentes, Alexandra Dostal, Amanda N. Payne, Pamela Vazquez Gutierrez, Christophe Chassard, Franck Grattepanche, Willem M. de Vos, Christophe Lacroix

**Affiliations:** 1 Institute of Food, Nutrition and Health, ETH Zurich, Zurich, Switzerland; 2 Laboratory of Microbiology, Wageningen University, Wageningen, The Netherlands; 3 Department of Basic Veterinary Medicine, University of Helsinki, Finland; Instutite of Agrochemistry and Food Technology, Spain

## Abstract

*In vitro* gut fermentation modeling offers a useful platform for ecological studies of the intestinal microbiota. In this study we describe a novel Polyfermentor Intestinal Model (PolyFermS) designed to compare the effects of different treatments on the same complex gut microbiota. The model operated in conditions of the proximal colon is composed of a first reactor containing fecal microbiota immobilized in gel beads, and used to continuously inoculate a set of parallel second-stage reactors. The PolyFermS model was validated with three independent intestinal fermentations conducted for 38 days with immobilized human fecal microbiota obtained from three child donors. The microbial diversity of reactor effluents was compared to donor feces using the HITChip, a high-density phylogenetic microarray targeting small subunit rRNA sequences of over 1100 phylotypes of the human gastrointestinal tract. Furthermore, the metabolic response to a decrease of pH from 5.7 to 5.5, applied to balance the high fermentative activity in inoculum reactors, was studied. We observed a reproducible development of stable intestinal communities representing major taxonomic bacterial groups at ratios similar to these in feces of healthy donors, a high similarity of microbiota composition produced in second-stage reactors within a model, and a high time stability of microbiota composition and metabolic activity over 38 day culture. For all tested models, the pH-drop of 0.2 units in inoculum reactors enhanced butyrate production at the expense of acetate, but was accompanied by a donor-specific reorganization of the reactor community, suggesting a concerted metabolic adaptation and trigger of community-specific lactate or acetate cross-feeding pathways in response to varying pH. Our data showed that the PolyFermS model allows the stable cultivation of complex intestinal microbiota akin to the fecal donor and can be developed for the direct comparison of different experimental conditions in parallel reactors continuously inoculated with the exact same microbiota.

## Introduction

The human colon is the most densely colonized part of the digestive tract harboring a diverse, host-specific consortium of microorganisms that accounts for approx. 2% of the body mass [1]. It is home to 10^11^–10^12^ bacteria per gram of contents belonging to an estimated 1800 genera and 15′000–36′000 different species as revealed by culture-independent metagenomic surveys [2], [3], [4]. This microbial community decisively contributes to morphological, immunological and nutritional functions of the digestive tract and may be involved in many diseases [5], [6], thus directly acting on human health. Hence, there is considerable interest in exploring the overwhelming enzymatic and metabolic functions of the colonic microbiota that are directly linked to commensal community structure and are highly influenced by various exogenous factors (e.g. diet, drugs, chronic and acute diseases). To unravel this complex interplay, system biology approaches combining *in vitro* and *in vivo* models with high-throughput molecular and state-of-the-art ‘-omics’ technologies may be recommended [7].

In this context, *in vitro* gut fermentation models represent a useful, yet host-uncoupled tool for compositional and functional studies highly challenged in humans and animals owing to ethical concerns and hindered accessibility of intestinal contents [8]. Different types of colonic *in vitro* models are currently in use, ranging from simple batch [9], [10] and more complex single- [11], [12] or multistage (SHIME, [13]) continuous or semi-continuous cultures, to artificial models accounting for metabolite and water absorption (TIM-2, [14]). Common to all models is the aim of stable cultivation an intestinal microbiota for a defined period of time while preserving the activities of the predominant microbial groups. While batch models are limited to short-term fermentation experiments, continuous systems can be operated for longer periods of typically 2–4 weeks under pseudo-steady state conditions. However, most *in vitro* fermentation models are inoculated with a liquid fecal suspension resulting in a fragile community establishment due to the lack of biofilm-associated states of bacterial populations in conjunction with a continuous wash-out of less competitive bacteria [15], [16]. To address this issue, an immobilization process for the entrapment of fecal microbiota in mixed xanthan-gellan gum gel beads was developed to maintain the microbial diversity over long time continuous colonic fermentations and reach similar high cell densities to the colon [7], [17]. The use of immobilized fecal microbiota allows creating self-contained continuous fermentation systems characterized by long-term functionality [18], [19], [20], [21], [22], [23], [24], [25]. However, reproducibility and functional stability of the microbiota in gut fermentation models is often questioned but, together with true biological replicates, constitute a prerequisite for generating robust data. Furthermore, the comparison of the effect of treatments with the same microbiota is difficult to achieve because the microbiota is subject to temporal modification in continuous culture and large inoculum quantities are needed for batch cultures which must also be repeated [7].

In this study, a novel Polyfermentor Intestinal Model design (PolyFermS) was developed aiming at circumventing problems of reproducibility as well as biological replication, and allowing testing in parallel the effects of different treatments on the same complex gut microbiota. Effluents of the first-stage continuous inoculum reactor (IR) containing immobilized fecal microbiota and mimicking the upper proximal colon were used to continuously feed a set of second-stage control (CR) and test (TR) reactors operated in parallel with conditions of the proximal colon. To compensate for initial metabolic imbalances observed in IR and to more closely mimick conditions of high metabolic activity of the upper proximal colon, the pH set-point in inoculum reactors was decreased 0.2 units from 5.7 to 5.5 after 13 days. Three independent replicates inoculated with feces from different child donors were carried out (models A to C). Control reactors were used to study microbial composition and metabolic stability of models over time, while up to three test reactors were used to assess intra-system reproducibility. The microbial diversity of fecal inocula from the three donors was compared to reactor effluent samples using the Human Intestinal Tract Chip (HITChip), a high-density microarray that consists of over 5′000 oligonucleotide probes targeting 16S rRNA gene sequences of over 1′100 phylotypes of the human gastrointestinal tract [26]. Our results highlight the benefits of the novel PolyFermS model design allowing a stable and reproducible cultivation of complex intestinal communities in multiple reactors that can be used to simultaneously study the effects of several conditions (environmental parameters, dietary compounds, drugs, added microbes, etc.) compared to a control reactor.

## Materials and methods

### Ethics Statement

This work was approved by the Ethics Committee of ETH Zurich, Zurich, Switzerland (EK 2009-N-01). Informed written consent was obtained from parents of fecal donors and the children assented to the study.

### Fecal Sample Immobilization and Fecal Beads Colonization

Fecal samples (ca. 5 g) collected from three healthy donors (A: 6 year-old, male; B: 10 year-old, female; C: 8 year-old, male) receiving a fully diversified diet were transferred to a tube containing 25 mL of sterile, pre-reduced peptone water (0.1%, pH 7), placed in an anaerobic jar (Anaerojar, Oxoid, Hampshire, England) and immediately delivered to the laboratory. None of the children had been exposed to antibiotic treatment for three months prior to experimentation. Immobilization in 1–2 mm diameter gel beads composed of 2.5% gellan gum, 0.25% xanthan gum and 0.2% sodium citrate (w/v, Sigma-Aldrich Chemie GmbH, Buchs, Switzerland) was carried out as described previously [24]. The entire process was performed in an anaerobic chamber within 3 h after defecation and 60 ml fresh fecal gel beads from each donor were immediately transferred to inoculum reactors (IR, Sixfors, Infors, Bottmingen, Switzerland) of models A, B and C (IRA IRB and IRC) containing 140 ml nutritive medium (working volume: 200 ml). Beads were colonized for 48 h during batch cultures with conditions of the child proximal colon (T = 37°C; pH 5.7, control with addition of 2.5 N NaOH, continuous flow of pure CO_2_ in the reactor headspace). The fermented medium was replaced every 12 h with fresh nutritive medium.

### Nutritive Culture Medium

A complex culture medium mimicking the intestinal chyme of a child was used. The medium was similar to that described by Macfarlane *et al.* [27] modified for children by reducing the bile salt concentration from 0.4 to 0.05 g/l [28]. A volume of 0.5 ml of a filter-sterilized (Minisart, 0.2 µm pore-size, Sartious, Göttingen, Germany) vitamin solution [29] was added to 1 l of the autoclaved (15 min, 121°C) medium.

### Operation Conditions of the PolyFermS Model

**Figure 1:**
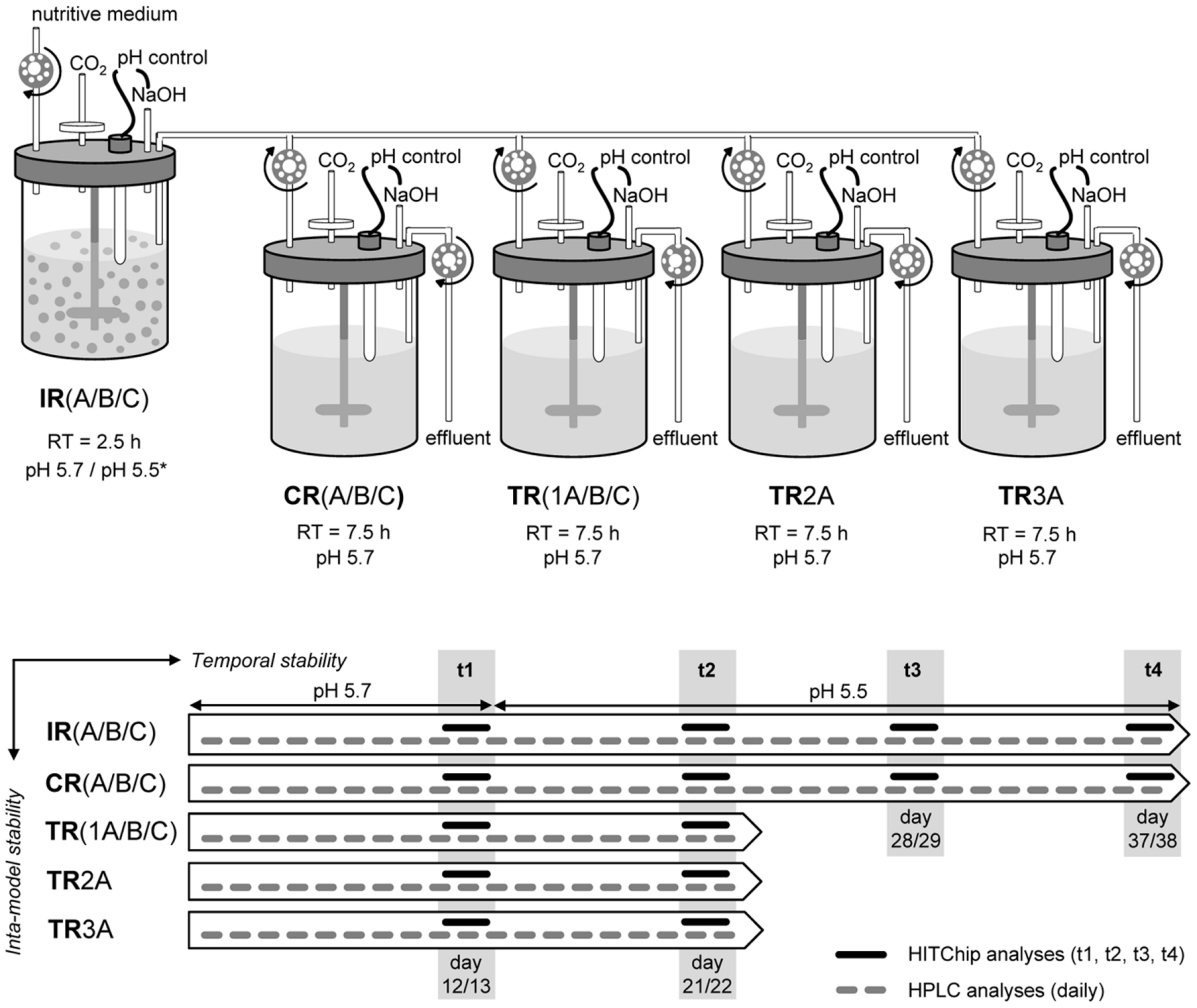
Setup and design of the Polyfermentor Intestinal Model (PolyFermS). Control (CR) and test reactors (TR) were continuously inoculated with effluents from inoculum reactors (IR) containing 30% (v/v) of fecal beads from donors A (model A), B (model B) and C (model C), respectively. Metabolites were quantified daily by HPLC analysis. Community structure was studied by HITChip analyses at selected time points t1, t2, t3 and t4. RT, mean retention time.

Continuous fermentation was started by respectively connecting each inoculum reactors IRA, IRB and IRC to control reactors (CRA, CRB and CRC) and from one to three test reactors (model A: TRA1, TRA2, TRA3; model B: TRB; model C: TRC; Figure 1) mounted in parallel and half-filled with sterile nutritive medium (37°C). Fresh sterile nutritive medium (4°C) was pumped continuously via peristaltic pumps (Reglo analog, Ismatec, Glattbrugg, Switzerland) to IRA, IRB and IRC at a feed flow rate of 80 ml/h (mean retention time of 2.5 h) and fermented effluents were equally distributed in the second-stage CR and TR of the model, with working volumes of 300 ml for a mean retention time of 7.5 h. Fermented medium from all reactors was pumped to an effluent receiving vessel. Operational parameters were chosen to mimic physiological condition of the upper (IR) and child proximal colon (CR and TR), with an overall retention time of 10 h (Figure 1). The pH was automatically controlled at 5.7 by adding 2.5 N NaOH during the first 12 days and was decreased thereafter to pH 5.5 in IR after 13 days to account for initial metabolic imbalances and account for a likely lower pH in this section of the colon with high microbial activity. All reactors of models A, B and C were operated in parallel for a total of 38 days. Control reactors served as intra-model control for assessing system stability. Test reactors were used to study intra-system stability until day 22 and were further operated for testing different experimental conditions (data not reported).

### Sampling

Effluent samples (10 ml) from all reactors were collected daily and processed within 1 h for quantification of short-chain fatty acids (SCFA: acetate, propionate and butyrate) by high-performance liquid chromatography (HPLC). Effluent samples were also taken on days 12/13 (t1) and 21/22 (t2) from all reactors, and on days 29/30 (t3) and 37/38 (t4) from inoculum (IR) and control reactors (CR) for phylogenetic profiling of bacterial communities by HITChip analysis.

### Metabolic Activity Analysis

Short-chain fatty acids (SCFA: acetate, propionate and butyrate) concentrations in effluent samples obtained from days 6 to 22 from all reactors and from days 23 to 38 from inoculum (IR) and control (CR) reactors were determined in duplicate by high-performance liquid chromatography (HPLC) analysis as described previously [24].

### HITChip Microarray Analysis

The HITChip phylogenetic microarray contained over 5′000 specific oligonucleotide probes targeting the V1 and V6 hypervariable regions of the 16S rRNA gene obtained from >16′000 human intestinal sequences, grouped into 27 order-like level-1, 131 genus-like level-2 and 1′140 unique phylotypes level-3 groups [26], [30]. HITChip analyses were performed as described by Rajilic-Stojanovic *et al.* [26] with samples obtained at four consecutive time points during continuous fermentation: t1, days 12/13 (all reactors before IR pH-switch); t2, days 21/22 (all reactors after IR pH-switch); t3, days 29/30 (IR and CR for time stability); t4, days 37/38 (IR and CR for time stability) (Figure 1). Briefly, genomic DNA was extracted from a 1∶1 mix of 1.5 ml effluent sample with the Fast DNA Spin Kit for Soil (MP Biomedicals, Illkirch, France) according to the manufacturer’s instructions with a final elution volume of 100 µl. The full-length 16S rRNA gene was amplified and PCR products were transcribed into RNA before being labeled with Cy3 and Cy5, fragmented and hybridized in duplicates on the microarray. The microarrays were scanned with an Agilent DNA Microarray Scanner. Data were extracted from images using the Agilent Feature Extraction software version 10.7.3.1, normalized and further analyzed using a set of R-based scripts (http:www.r-project.org) in combination with a custom-designed database that runs under MySQL management system as described elsewhere [26], [31].

### Statistical Analysis

The similarity of microbial profiles obtained for fecal donor samples and reactor effluents using the HITChip microarray was assessed by calculating Pearson’s product-moment correlation (Pearson’s correlation) and the Ward’s minimum variance method was used for the generation of hierarchical clustering of probe profiles [32]. The diversity of the microbiota in reactor effluents and donor samples was calculated with the Simpson’s reciprocal index of diversity (1/D), with a higher value corresponding to a more diverse community. Intra-model reproducibility of metabolic activity was assessed by calculating Pearson’s product-moment correlation (Pearson’s correlation) coefficients for acetate, propionate and butyrate ratios in CR and TR of the same model measured from day 6 to 22. A Wilcoxon signed-rank test performed (JMP 8.0 for Windows, SAS Institute Inc., Cary, NC, USA) was used for evaluating the statistical significance of observed differences between individual phylogenetic groups measured by HITChip analyses of effluent samples collected from all reactors of the same model before (t1; model A: n = 5, model B and C: n = 3) and after (t2; model A: n = 5, model B and C: n = 3) the pH-switch. The statistical significance of observed differences between mean metabolite concentrations and ratios measured by HPLC were evaluated for effluent samples collected from all reactors of the same model before (day 12 and 13, model A: n = 10, model B and C: n = 3) and after (day 21 and 22, model A: n = 10, model B and C: n = 3) the pH- switch. Values were considered significant at *P*<0.05.

## Results

### Metabolic Activity and Microbial Profiles for pH 5.7 in IR

**Figure 2:**
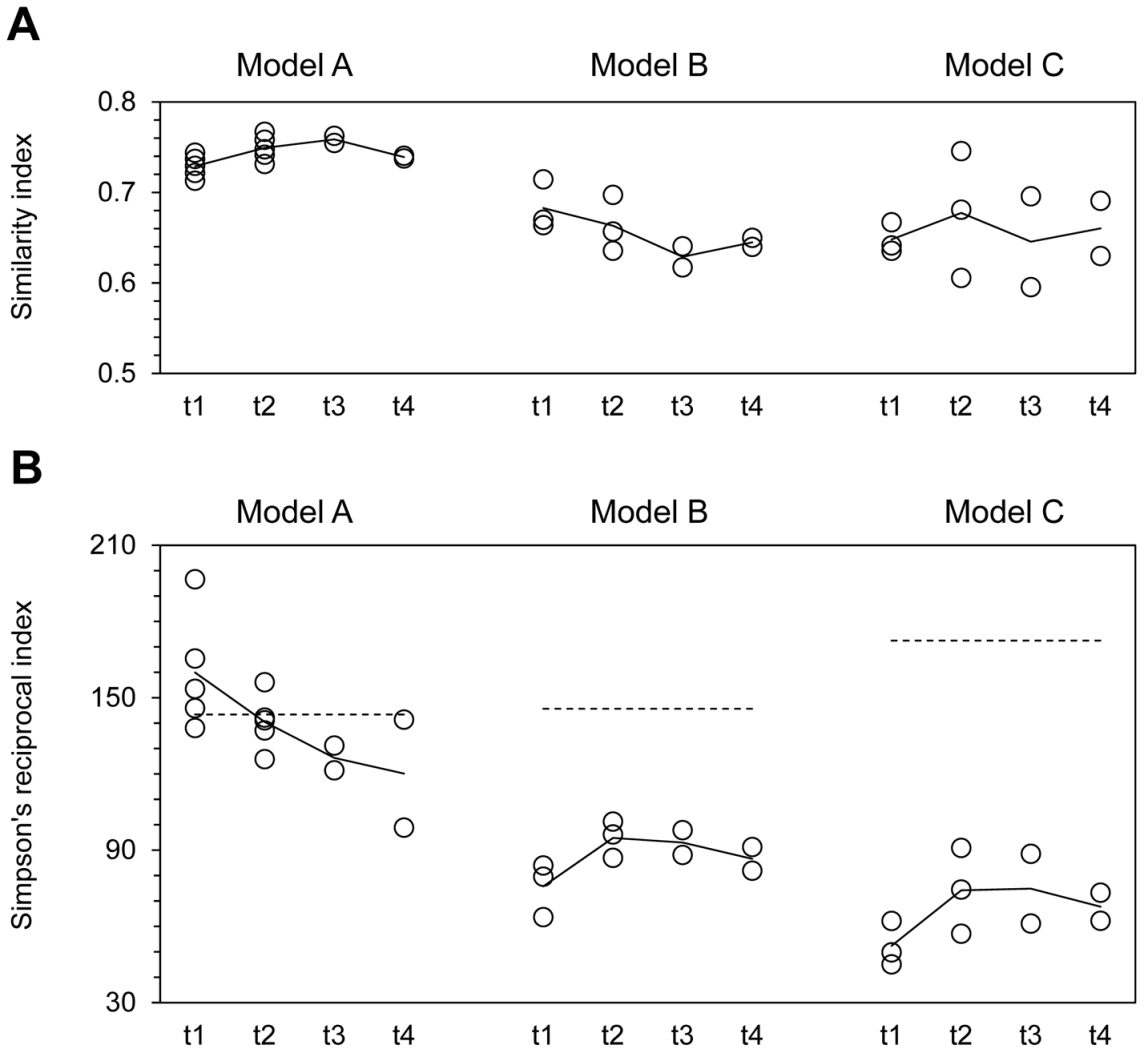
Composition of intestinal microbiota produced in effluents of PolyFermS models. **A. Similarity to the fecal donor.** Open circles and straight lines correspond to single and mean similarity indices, respectively, based on Pearson product-moment correlation coefficients for HITChip fingerprints generated from fecal donor samples and PolyFermS model reactor effluents obtained from model A, B and C at time points t1 and t2 from all reactors and at t3 and t4 from IR and CR. **B. Temporal diversity development.** Open circles and lines correspond to single and mean Simpson’s reciprocal indices of diversity, respectively, calculated for effluent samples obtained from model A, B and C at time points t1 and t2 from all reactors and at t3 and t4 from IR and CR. Dashed lines indicate the Simpson’s reciprocal index of the corresponding fecal donor sample. A higher Simpson’s reciprocal index reflects a more diverse community, e.g. in terms of species richness and evenness.

For all three models, the mean metabolic activity in IR, CR and TR of the PolyFermS model was very high during the first 13 days of continuous culture when the pH in IR was set at 5.7 (total SCFA concentration in model A: 199±14 mM, model B: 188±18 mM, model C: 174±16 mM; Table 1). The molar acetate/propionate/butyrate ratios in model A (77%/8%/15%, Table 2) differed from model B (66%/20%/14%) and C (61%/26%/14%). The microbiota composition in all models after days 12/13 (t1) showed differences with corresponding donors’ fecal microbiota, with mean similarity indices of 0.73±0.01, 0.68±0.03 and 0.65±0.02 for model A, B and C, respectively (Figure 2A). Model-specific changes were detected for the most important higher taxonomic groups (Table 2, Table S1, and Table S2). For example, the mean relative abundance of Bacteroidetes was higher in models A (32.6%) and B (32.5%) but equal in model C (37.4%) compared to the corresponding fecal donor (A: 6.3%, B: 18.3%, C: 36.7%). The abundance of Firmicutes in reactor effluents of model A (50.6%) and B (62.1%) was slightly lower compared to the fecal donors (A: 79.3%, B: 80.7%), with *Clostridium* cluster XIVa and IV being most decreased in model A and B, respectively. In model C, total Firmicutes established at similar high levels (53.3%) to donor’s feces C (56.1%), but with intra-group shifts. A decrease of *Clostridium* cluster IV from 19.0% to 4.7% and an increase of *Clostridium* cluster XIVa from 31.7% to 46.4% was observed from donor to model C. Levels of *Actinobacteria* were similar for donor A (12.4%) and model A (11.6%), but increased from 0.4% to 4.8% from donor to model B and decreased from 6.2% to 2.4% from donor to model C. For all models, the transfer from *in vivo* to *in vitro* conditions resulted in higher levels of Proteobacteria in reactor effluents compared to donor samples.

**Table 1:** Metabolite concentrations and molar ratios in reactor effluents of models A, B and C before (t1) and after (t2) the pH-switch.

		PolyFermS model A		PolyFermS model B		PolyFermS model C	
		t1	t2	t1	t2	t1	t2
**Acetate**	(mM)	147 (±9)	91 (±22)*	111 (±14)	99 (±12)	98 (±10)	74 (±6)*
	(%)	77 (±1)	53* (±2)	66 (±4)	61* (±1)	61 (±1)	47* (±1)
**Propionate**	(mM)	15 (±3)	10 (±3)*	35 (±8)	36 (±5)	41 (±4)	46 (±4)
	(%)	8 (±1)	6* (±1)	20 (±3)	22 (±1)	26 (±1)	29* (±1)
**Butyrate**	(mM)	28 (±1)	73 (±16)*	24 (±5)	30 (±5)	22 (±4)	39 (±4)*
	(%)	15 (±1)	41* (±3)	14 (±1)	17* (±1)	13 (±1)	25* (±1)
**Total**	(mM)	199 (±14)	173 (±23)	188 (±18)	171 (±22)	174 (±16)	160 (±14)

All values are given as mean (±SD, for concentrations) calculated for all reactors of the same model before (t1, days 12 and 13; model A: n = 10, models B and C: n = 6) and after (t2, days 21 and 22; model A: n = 10, models B and C: n = 6) the pH-switch.

* Means of concentrations or ratios, respectively, differ significantly (*p*<0.05) for the same metabolite between t1 and t2.

**Table 2:** Mean relative abundance (%) of higher taxonomic groups in fecal samples of donors A, B and C and in reactor effluents of corresponding PolyFermS models A, B and C before (t1) and after (t2) the pH-switch.

			PolyFermS model A			PolyFermS model B			PolyFermS model C	
		Donor A	t1	t2	Donor B	t1	t2	Donor C	t1	t2
**Actinobacteria**		12.4	11.6 (±1.7)	10.7 (±3.2)	0.4	4.8 (±1.4)	3.5 (±1.3)	6.2	2.4 (±1.2)	2.2 (±1.1)
**Bacteroidetes**		6.3	32.6 (±7.9)	23.5 (±8.4)	18.3	32.5 (±4.1)	30.1 (±2.9)	36.7	37.4 (±4.6)	38.6 (±5.0)
**Firmicutes**		79.3	50.6 (±7.1)	58.8 (±9.1)	80.7	62.1 (±4.5)	55.3 (±3.1)	56.1	53.3 (±3.3)	52.7 (±1.0)
	Bacilli	1.1	1.6 (±1.6)	2.9 (±1.3)	0.5	0.3 (±0.1)	0.4 (±0.1)	0.7	0.5 (±0.1)	0.8 (±0.2)
	*Clostridium* cluster I	0.2	<0.05	<0.05	0.1	<0.05	<0.05	0.7	1.1 (±1.4)	0.1 (±0.1)
	*Clostridium* cluster III	0.7	<0.05	<0.05	0.7	0.3 (±0.2)	0.4 (±0.3)	0.2	<0.05	<0.05
	*Clostridium* cluster IV	6.1	6.9 (±0.8)	6.2 (±2.9)	20.3	8.2 (±4.3)	7.3 (±4.0)	19.0	4.7 (±1.5)	15.6 (±1.6)
	*Clostridium* cluster IX	<0.05	0.2 (±0.1)	0.1 (±0.1)	0.3	0.2 (±0.1)	0.3 (±0.0)	0.1	0.2 (±0.1)	0.3 (±0.1)
	*Clostridium* cluster XI	3.2	0.7 (±0.2)	0.5 (±0.3)	0.6	0.4 (±0.1)	0.6 (±0.1)	2.7	0.2 (±0.1)	0.1 (±0.1)
	*Clostridium* cluster XIVa	67.4	41.1 (±7.6)	49.1 (±6.1)	56.8	52.4 (±0.4)	46.0 (±1.0)	31.7	46.4 (±3.1)	35.8 (±1.6)
	*Clostridium* cluster XVI	<0.05	<0.05	<0.05	<0.05	<0.05	<0.05	<0.05	<0.05	<0.05
	*Clostridium* cluster XVIII	0.8	<0.05	<0.05	0.1	0.1 (±0.0)	0.2 (±0.1)	0.6	0.1 (±0.0)	<0.05
	Uncultured *Clostridiales*	<0.05	<0.05	<0.05	1.3	0.2 (±0.1)	0.2 (±0.2)	0.6	<0.05	<0.05
**Proteobacteria**		1.7	5.0 (±0.9)	6.9 (±2.0)	0.1	0.6 (±0.4)	11.0 (±4.1)	0.3	6.8 (±0.7)	6.5 (±3.2)

Values are given as mean (±SD) calculated for the relative abundances of higher taxonomic groups based on HITChip analysis of fecal samples of donors (n = 1) and of reactor effluents obtained from all reactors of the same model before (t1, days 12/13; model A: n = 5, models B and C: n = 3) and after (t2; model A: n = 5, models B and C: n = 3) the pH-switch.

The diversity of the microbial community from donor samples was more conserved in model A compared to models B and C (Figure 2B). The Simpson’s index of diversity at t1 decreased from 146 (donor B) to 76±11(for model B reactors at days 12/13) and from 172 (donor C) to 52±9 for model C, but unexpectedly increased in model A from donor (143) to reactors at day 12/13 (160±23).

### Effects of Decreased pH in IR on Metabolic Activity and Microbial Composition

Metabolic activity for all models was very high when pH in IR was controlled at 5.7, with high reactor acetate concentrations and imbalanced SCFA ratios. The pH in inoculum reactors (IR) of all models was decreased by 0.2 units (from 5.7 to 5.5) from the 13^th^ day to decrease activity and more closely mimic low upper proximal colon pH. In all models, the pH-drop resulted in significantly lower acetate and higher butyrate molar ratios at t2 (days 21/22) compared to values recorded before the pH-change (Table 1). Furthermore, the lower pH resulted in a pronounced decrease of metabolic activity in all models, with total SCFA concentrations dropping by approximately 10% after the pH was decreased.

The microbial community was more complex in all reactors of models B and C when pH in IR was controlled at 5.5, with an increase of mean Simpson’s diversity index from 76±11 (t1) to 95±7 (t2) and from 52±9 (t1) to 74±17 (t2) for model B and C, respectively, before and after the pH change (Figure 2B). In contrast, the microbiota in model A became more similar to the fecal donor sample A (Figure 2A) accompanied by a slight diversity-drop, with mean Simpson’s diversity index decreasing from 160±23 (t1) to 140±11 (t2, Figure 2B) and significant changes observed for microbial profiles (Table 3). The mean relative abundance of members belonging to the Bacteroidetes group (e.g. *Bacteroides fragilis et rel.*, *Bacteroides intestinalis et rel.* or *Bacteroides vulgatus et rel.*) significantly decreased, while important butyrate producers belonging to the *Clostridium* clusters XIVa (e.g. *Eubacterium rectale et rel.* or *Roseburia intestinalis et rel.*) increased when pH was decreased.

**Table 3:** Mean relative abundance of phylotypes that were highly affected by the pH-switch in PolyFermS model A.

Higher taxonomic group	Phylotype	t1	t2	corrected *p* value
Bacteroidetes	*Bacteroides fragilis et rel.*	2.22	1.08*	0.037
	*Bacteroides intestinalis et rel.*	1.88	1.40*	0.022
	*Bacteroides vulgatus et rel.*	5.49	3.96*	0.022
	*Tannerella et rel.*	1.05	0.54*	0.012
*Clostridium* cluster XIVa	*Clostridium nexile et rel.*	2.30	3.26	0.060
	*Eubacterium rectale et rel.*	2.05	4.13	0.060
	*Roseburia intestinalis et rel.*	2.70	5.83	0.060
Proteobacteria	*Proteus et rel.*	0.02	0.08*	0.037
	*Sutterella wadsworthia et rel.*	3.36	1.89*	0.037

All values are given as mean relative abundances (%) of phylotypes based on HITChip analysis of reactor effluents obtained from all reactors of the PolyFermS model A before (t1, day 12/13; n = 5) and after (t2, day 21/22; n = 5) the pH-switch. A comprehensive overview of the most abundant phylotypes detected before and after the pH-switch is given in Table S3.

* Means with an asterisk differ significantly (*p*<0.05) for the same phylotype before and after the pH-switch.

### Correlation between Butyrate Metabolism and Community Structure

**Figure 3:**
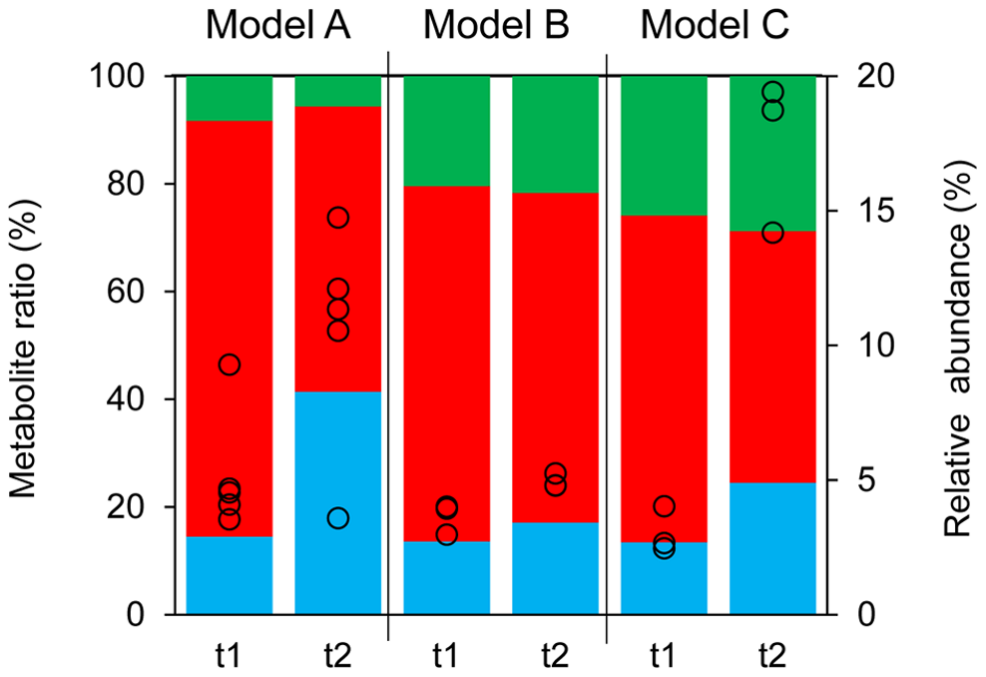
Correlation between butyrate production and the fraction of predominant butyrate-producing bacteria in the total microbiota produced in effluents of PolyFermS models. Open circles visualize the mean relative abundance (% of total flora) of predominant butyrate-producing bacteria (*Faecalibacterium prausnitzii, Eubacterium rectale, Roseburia spp.*) in IR, CR and TR of model A, B and C and colored bars indicate mean model-specific molar metabolite ratios (%) of acetate (in red), propionate (in green) and butyrate (in blue), with IR pH at 5.7 (t1) and 5.5 (t2).

A correlation between the butyrate ratio and the relative abundance of predominant butyrate-producing bacteria, including *Faecalibacterium prausnitzii* (*Clostridium cluster* IV), *Eubacterium rectale* and *Roseburia spp.* (*Clostridium cluster* XIVa), was observed for all reactors (IR, CR and TR) of models A and C as a response to the pH-switch (Figure 3). The pH-drop was associated with a significantly increased mean butyrate ratio from 15% to 42% and from 14% to 24% for model A and C, respectively (Table 2), correlating with increasing mean abundance of predominant butyrate-producing bacteria increasing from 5.2% to 10.5% and from 3.1% to 17.4%. In contrast, model B showed very limited change for the abundance of butyrate producers and butyrate production upon lower pH.

### Reproducibility and Temporal Stability of the PolyFermS Model

**Figure 4:**
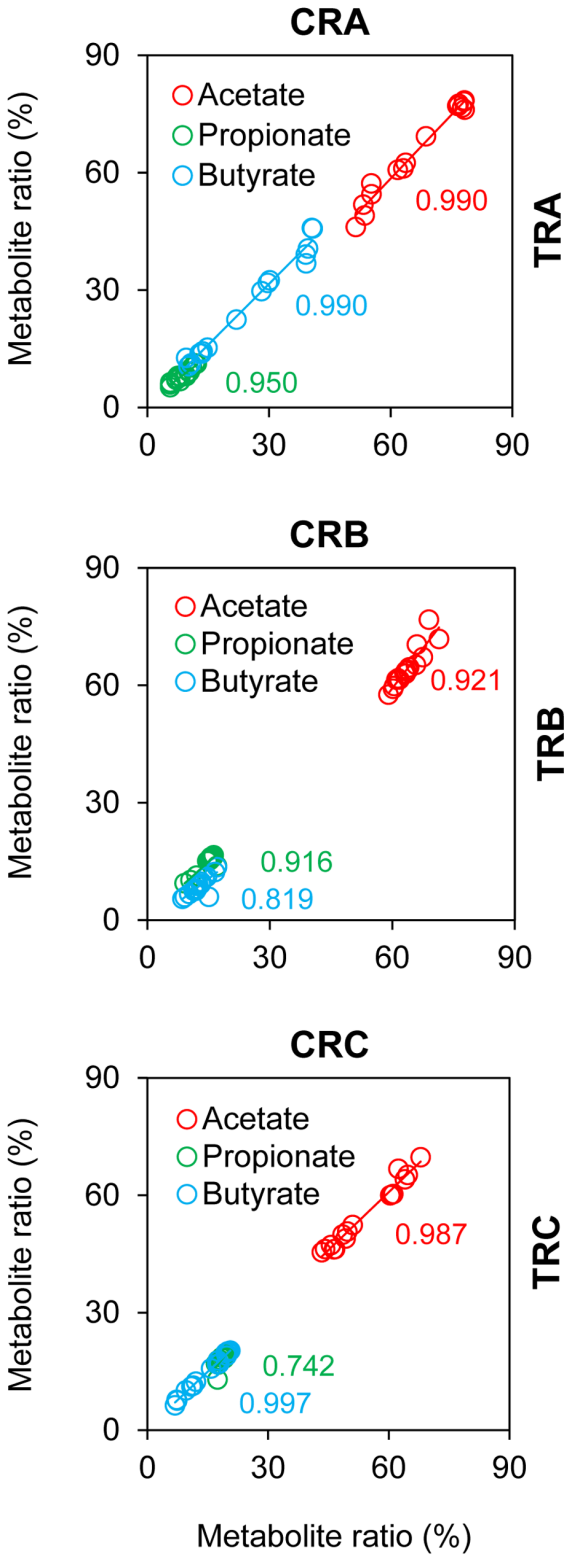
Intra-model reproducibility of metabolic balance measured in effluent samples of PolyFermS models. Molar metabolite ratios (%) measured daily in control reactors (CRA, CRB and CRB; x-axis) and test reactors (TRA, TRB and TRC; y-axis) from day 6 to 22. Mean daily ratios were calculated for TR1A, TR2A, TR3A (TRA). Numbers in color indicate model-specific Pearson correlation coefficients calculated for acetate (in red), propionate (in green) and butyrate (in blue).

**Figure 5:**
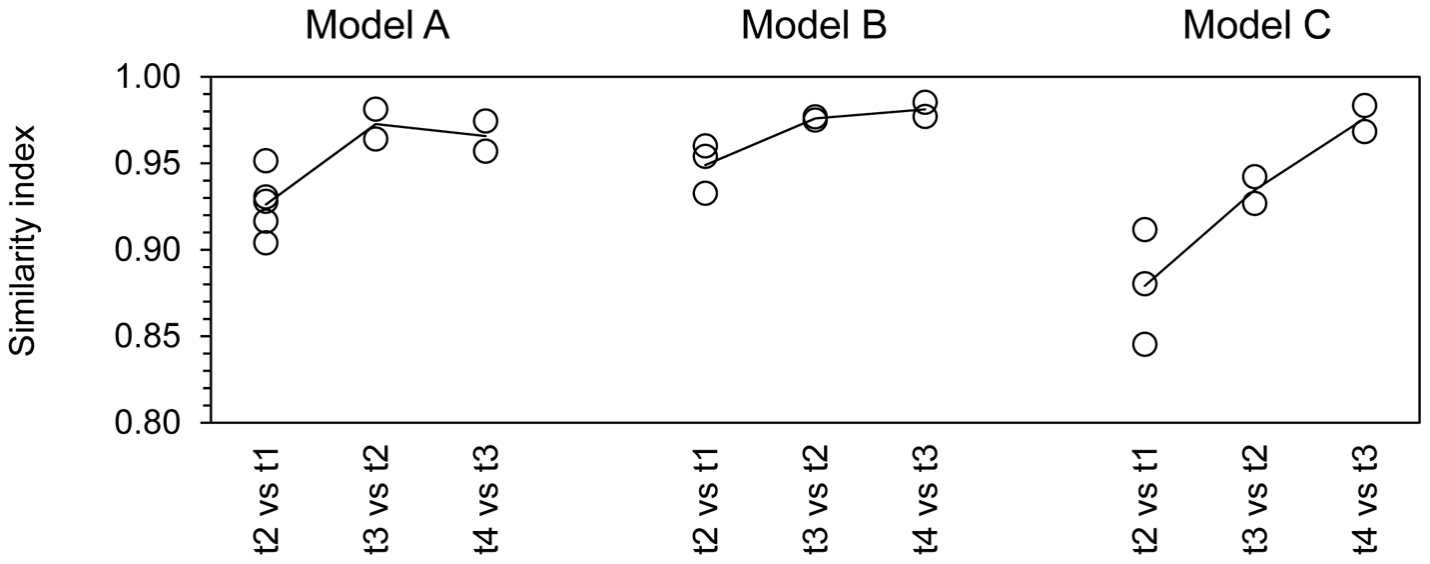
Time stability of intestinal microbiota produced in effluents of PolyFermS models. Single (open circles) and mean (line) similarity indices are based on Pearson product-moment correlation coefficients for HITChip fingerprints generated from PolyFermS reactor effluents obtained from model A, B and C at time points t1 and t2 from all reactors and at t3 and t4 from IR and CR compared to corresponding previous time points.

Metabolic activity was very stable in IR and CR from day 6 until the end of fermentation on day 38 (Figure S1, S2 and S3). Pearson correlation coefficients calculated for acetate, propionate and butyrate ratios between control (CR) and test (TR) reactors of the same model were close to 1 (Figure 4), except for propionate in model C (0.742). In addition, structural diversity was highly reproducible in all models, as revealed by Simpson’s reciprocal indices that were similar for all reactors (IR, CR and TR) of the same model at day 21/22 (t2) and for CR and TR at day 28/29 (t3) and at day 37/38 (t4; Figure 2B). For all models, similarity indices of HITChip fingerprints were high (>0.95) for IR and CR and for all models at day 37/38 (t4) compared to day 28/29 (t3; Figure 5). Metabolic profiles and ratios of Actinobacteria, Proteobacteria, Bacteroidetes and Firmicutes were very stable in IR and CR of all models after pH adjustment until the end of fermentation (Figure S1, S2 and S3).

## Discussion

### Establishment of an Immobilized Fecal Microbiota in in vitro Proximal Colon Conditions

The Human Intestinal Tract Chip (HITChip) used in this study is a high-resolution phylogenetic microarray that was previously used for an in-depth analysis of intestinal ecosystems to characterize the intestinal community development in *in vitro* gastrointestinal simulators (SHIME, TIM-2) [13], [32]. Irrespective of bacterial profiles in fecal donor samples, Actinobacteria/Bacteroidetes/Firmicutes ratios measured in PolyFermS model of the proximal colon (Table 1) were within the range usually reported for fecal microbiota of healthy children and adults (8–17/10–30/46–58%, respectively, [33], [34]). In contrast, HITChip fingerprints of samples obtained from the SHIME model [13] showed low ratio of Actinobacteria (0.7%) and Firmicutes (13.2%) and high ratio of Bacteroidetes (75.6%). Similar shifts were reported for the TIM-2 model of the large intestine analyzed with HITChip, with higher Bacteroidetes and lower Firmicutes ratios compared to fecal inocula from various volunteers [32]. One important characteristic of PolyFermS model is the use of immobilized fecal microbiota to reproduce both, planktonic and sessile states of bacterial populations in the colon and promote the retention of slow growing microbes and microbial diversity. Gel beads kept in the IR reactors create mucosal-like adhesion sites which are important for prolonged and stable colonization, in a protective, oxygen-depleted microenvironment [35]. The constant release of intestinal bacteria growing within beads close to the bead surface leads to stable colonization of the system [7], [17].

The liquid phase communities and the absence of adhesion sites were previously identified as important factors for decreased Firmicutes and increased Bacteroidetes ratios compared to *in vivo* intestinal environments [13], [32]. *Bacteroides* spp. are less adhesive than other members of the gut microbiota [36] and *in vitro* environments where mucus adhesion is missing may therefore provide a selective advantage to this group compared to highly adhesive microbes [13]. Furthermore, *Bacteroides* spp. could be favored in *in vitro* systems which could have a higher redox potential exceeding normal physiological levels decreasing Firmicutes. Our data showed that the use of immobilization circumvented shifts in Firmicutes and Bacteroidetes ratio often reported in *in vitro* conditions, with only limited (model A and B) or no change of ratios (model C) compared to fecal donors (Table 2). Interestingly, adaptation of major groups within Firmicutes to the *in vitro* environment was model-dependent (Table 2). *Clostridium* Cluster IV was lower in models B and C than in corresponding feces, but unaffected in model A, while *Clostridium* cluster XIVa was lower in model A, stable in model B and increased in model C. These different responses could reflect adaption to the new environment (simulated proximal colon vs. feces) which depends on conditions prevailing in the host at the time point of fecal sampling. The lack of host-related factors (e.g. water and metabolite absorption, intestinal cells) and the process of immobilization itself may also influence microbiota development.

### Relationship between Metabolic Activity and Microbiota Composition

The colonic microbiota produces an estimated amount of 100–450 mM of total SCFA daily through fermentation of unabsorbed carbohydrates. SCFA can be absorbed by the colonic epithelium and transported to peripheral tissues, with only 5–10% being excreted in feces [37]. Acetate is the main SCFA produced in the colon, followed by propionate and butyrate with molar ratios in the range of 60–80∶ 14–22∶ 8–23 in healthy people [38]. These weak acids impact gut microbiota composition and influence host health, with butyrate representing the preferred energy source for colonocytes [39]. Fermentative activity in inoculum reactors (IR) of the PolyFermS model operated with a very short mean retention time of only 2.5 h and high supply of nutritive medium at pH 5.7 was very high and indicated a shift toward acetate production for all models (Table 1). The abundant SCFA production combined with lack of absorption may create a restrictive environment for the growth of certain microbes most affected by the inhibitory effects of SCFA which increase at low pH. *Clostridium* clusters XIVa and IV contain important metabolic clades of broad fermentation potential and represent dominant SCFA producers of the gut microbiota. Differences between *Clostridium* cluster XIVa and IV in adapting to the low pH (5.7) suggest variable species-species adaptive capacities and a potential struggle between intrinsic regulatory factors and nutrient availability in determining community growth and metabolic activity [19].

### Effects of pH on Microbiota Composition and Metabolic Activity

The colonic pH plays an important role in controlling the gut colonization. It is generally lower in the proximal (5.6–5.9) compared to the distal (6.6–6.9) colon as a result of active fermentation of dietary substrates and the production of SCFA [37], [40], and may fluctuate between hosts depending upon dietary intake and physiological characteristics. Duncan *et al.* [12], [41] showed that large pH-shifts in continuous flow fermentor studies highly influenced gut microbiota composition. Growth of *Roseburia* spp. and *Eubacterium rectale* was promoted at pH 5.5 compared to 6.5 at the expense of *Bacteroides* spp. and associated with increased butyrate formation, in agreement with our data. We showed that a pH shift of only minus 0.2 pH-units induced a fast metabolic and ecological response (Figure S1), with decreased Bacteroidetes and increased Firmicutes ratios in model A, and enhanced butyrate at the expense of acetate in model A and C. *Clostridium* clusters XIVa (family *Lachnospiraceae*) and IV (family *Ruminococcaceae*) of Firmicutes are known to harbor many different species of butyrate-producing bacteria [42], [43], with *Roseburia* spp., *Eubacterium rectale* (both cluster XIVa) and *Faecalibacterium prausnitzii-*related bacteria (cluster IV) being particularly abundant at levels of approx. 2–15% of the total microbiota [44].

*In vitro* models allowed for a detailed assessment of pH effects according to the individual microbiota. For model C, the less pronounced pH response could be explained by differences in the equilibrium of C3 (propionate)/C4 (butyrate) fermentation end products, with molar ratios being more balanced in model C (54%/46%) compared to model A (12%/88%). Indeed, in addition to *Clostridium* cluster IX and Propionibacteria, the Bacteroidetes population has a broad saccharolytic capacity and is proficient at producing propionate from degradation of a wide range of polysaccharides including starch [45]. Bacteroidetes accounted for 38.6% of the total microbiota in model C but only 23.5% in model A, which may partly explain the higher propionate production tested in model C. Many bacteria in the colon survive by cross-feeding of breakdown products from degradation of complex carbohydrates or fermentation end products such as lactic acid [39]. Together with *Eubacterium hallii*, *Anaerostipes caccae* is an important lactate-utilizing gut bacteria that produce butyrate as a major fermentation product [46]. Indeed, a decrease of *A. caccae* was observed in model C but not in model A after the pH decrease, which may explain the higher production of butyrate in model A. Furthermore, butyrate formation arising from lactate cross-feeding pathways with *Bifidobacterium adolescentis* (lactate producer) and *E. hallii/A. caccae* (lactate utilizers) or acetate cross-feeding pathways with *Roseburia* and *A. caccae* are well documented [47], [48]. *Bifidobacterium* populations were much more abundant in model A (representing 10.1% of total microbiota) compared to model C (1.9%) after the pH-drop. It can therefore be assumed that different butyrate production pathways, induced by intermediate metabolite availability or small fluctuations in pH known to affect the conversion of lactate by the human intestinal microbiota [49] impacted on butyrate accumulation in different models.

The growth of Gram-negative Proteobacteria is normally inhibited under simulated gut conditions by reduced pH due to amended inhibitory effects of SCFAs [50]. Unexpectedly, the pH-decrease in model B resulted in a large increase of Proteobacteria (from 0.6% to 11%), mainly limited to potentially pathogenic serotypes of *Escherichia coli*, some of which possibly being more pH-tolerant than others. On the other hand, acetate, detected at higher concentrations in model B after the pH-drop compared to the other models (Table 1), is known to exert a strong inhibitory activity against Gram-negative bacteria [51]. With a pKa value of 4.76 however, it is highly deprotonated and weakly active at pH 5.5. The large increase of *E. coli et rel.* after the pH-drop may therefore be related to the absence of *Lactobacillus plantarum et rel.* in model B compared to model A (representing 2.4% of total microbiota) and C (0.4%), as many *L. plantarum* strains are known to produce bacteriocins with a broad inhibitory activity against Gram-positive and Gram-negative pathogenic bacteria including *E. coli* [52]. Overall, our data showed a consistent general response to pH for all three models inoculated with feces derived from different donors. However, individual effects on microbiota balance and activity related to a specific treatment tightens the need of repeating *in vitro* intestinal fermentations inoculated with feces from different donors, and of a throughout interpretation of effects and mediated mechanisms.

### Reproducibility and Temporal Stability of PolyFermS

The PolyFermS model enabled parallel operation of multiple test reactors (TR) continuously inoculated with the same microbiota produced in IR. This design allows parallel testing of different treatments compared to a control reactor (CR) where no treatment is applied [18] when ecological and metabolic characteristics are reproducible and similar in control (CR) and test (TR) reactors. In this study, metabolic steady-state-conditions were reached after approx. 6 days of continuous culture in all models. Remarkably high intra-model stability was observed between t3 and t4 (mean intra-model Pearson correlation, r = 0.97; standard deviation, ±0.01) similar to the temporal stability (<1 year) reported for the human intestinal microbiota of healthy adults [53], [54]. Furthermore, reproducibility of metabolic activity and phylogenetic fingerprints were generally high (Figure 5, Figure S1, S2 and S3), supporting this unique feature of the PolyFermS model for comparison of different treatments. The robustness of *in vitro* intestinal fermentation models highly depends on the certainty that any observed responses of the reactor community are only due to the applied experimental treatment and not to adaption to the simulated environment [7].

### Conclusions

The present study describes the validation of a novel colonic fermentation model design, PolyFermS, for ecological and metabolic studies of the gut microbiota. Compared to other intestinal fermentation models, this model is characterized by the advantageous possibility to stably and reproducibly cultivate complex intestinal communities in multiple reactors allowing studying in parallel the impact of many different treatments (environmental parameters, dietary compounds, drugs, added microbes, etc.) compared to a control reactor. This set-up can be extended to increase the number of second stage parallel reactors and to attach multistage systems to IR to mimic different sections of the colon in parallel systems seeded with the exact same microbiota produced in IR [18]. Using PolyFermS model, we could show a strong effect of pH on microbiota profiles and linked metabolic activities which depended on the donor microbiota. Our data support that individual microbiota derived from a single donor should be used to inoculate *in vitro* models to gather reliable data on whole ecosystem dynamics in response to a manipulated factor.

## Supporting Information

Figure S1 **Microbiota composition and metabolic activity of the PolyFermS model A over time.** Open circles visualize daily concentrations (mM) of acetate (in red), propionate (in blue) and butyrate (in green) in reactor effluents of IRA and CRA. Colored bars correspond to ratios (%) of Firmicutes (in green), Bacteroidetes (in red), Actinobacteria (in blue) and Proteobacteria (in black) detected in donor sample A and reactor effluents obtained at time points t1, t2, t3 and t4 from inoculum reactor IRA and control reactor CRA.
(TIF)

Figure S2 **Microbiota composition and metabolic activity of the PolyFermS model B over time.** Open circles visualize daily concentrations (mM) of acetate (in red), propionate (in blue) and butyrate (in green) in reactor effluents of IRB and CRB. Colored bars correspond to ratios (%) of Firmicutes (in green), Bacteroidetes (in red), Actinobacteria (in blue) and Proteobacteria (in black) detected in donor sample B and reactor effluents obtained at time points t1, t2, t3 and t4 from inoculum reactor IRB and control reactor CRB.
(TIF)

Figure S3 **Microbiota composition and metabolic activity of the PolyFermS model C over time.** Open circles visualize daily concentrations (mM) of acetate (in red), propionate (in blue) and butyrate (in green) in reactor effluents of IRC and CRC. Colored bars correspond to ratios (%) of Firmicutes (in green), Bacteroidetes (in red), Actinobacteria (in blue) and Proteobacteria (in black) detected in donor sample C and reactor effluents obtained at time points t1, t2, t3 and t4 from inoculum reactor IRC and control reactor CRC.
(TIF)

Table S1 **Normalized hybridization signal intensity for all 129 Level 2 (genus-like) phylogenetic groups targeted by the HITChip.**
(XLS)

Table S2 **Normalized hybridization signal for all 21 Level 1 (phylum-like) phylogenetic groups targeted by the HITChip.**
(XLS)

Table S3 **Relative abundance of phylotypes in PolyFermS model A before (t1) and after (t2) the pH-switch.**
(XLS)
