# Supplementary figures and images for: Novel Polyfermentor Intestinal Model (PolyFermS) for Controlled Ecological Studies: Validation and Effect of pH
Source: PLoS One. 2013 Oct 30;8(10):e77772. doi: 10.1371/journal.pone.0077772 (PMC3813750; doi:10.1371/journal.pone.0077772)

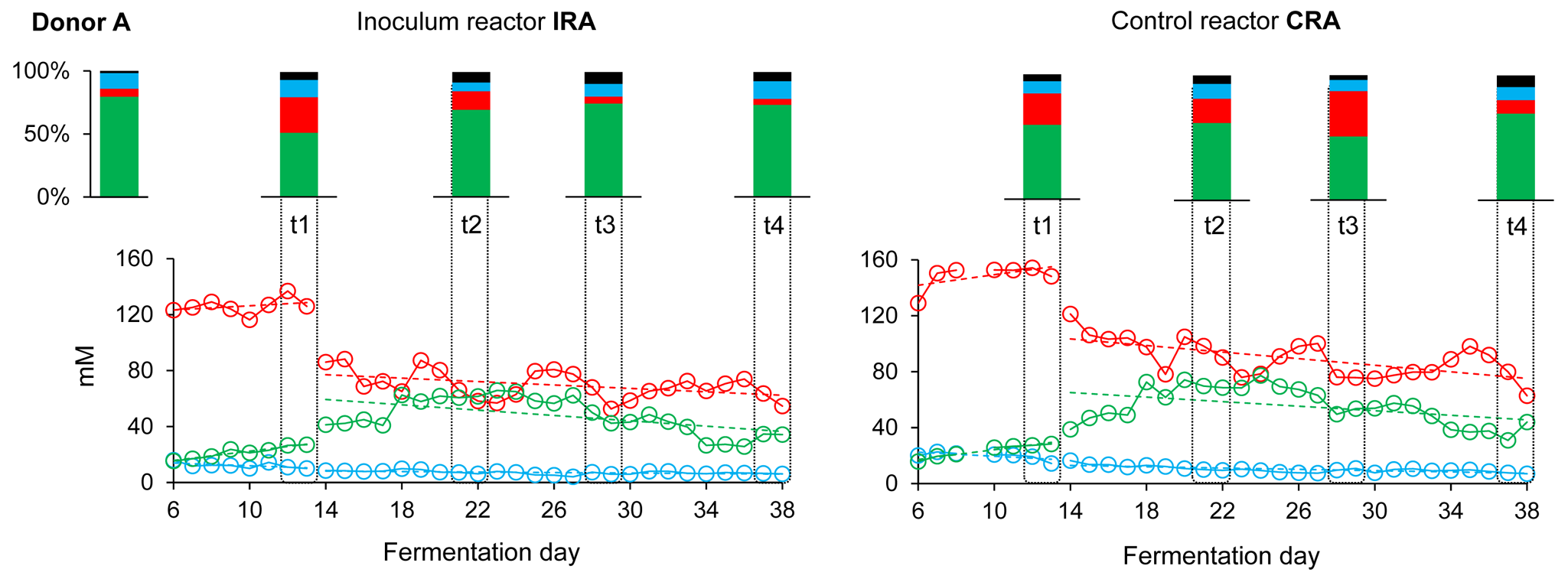

Supplement: Figure S1 — Microbiota composition and metabolic activity of the PolyFermS model A over time. Open circles visualize daily concentrations (mM) of acetate (in red), propionate (in blue) and butyrate (in green) in reactor effluents of IRA and CRA. Colored bars correspond to ratios (%) of Firmicutes (in green), Bacteroidetes (in red), Actinobacteria (in blue) and Proteobacteria (in black) detected in donor sample A and reactor effluents obtained at time points t1, t2, t3 and t4 from inoculum reactor IRA and control reactor CRA. (TIF) [file pone.0077772.s001.tif]

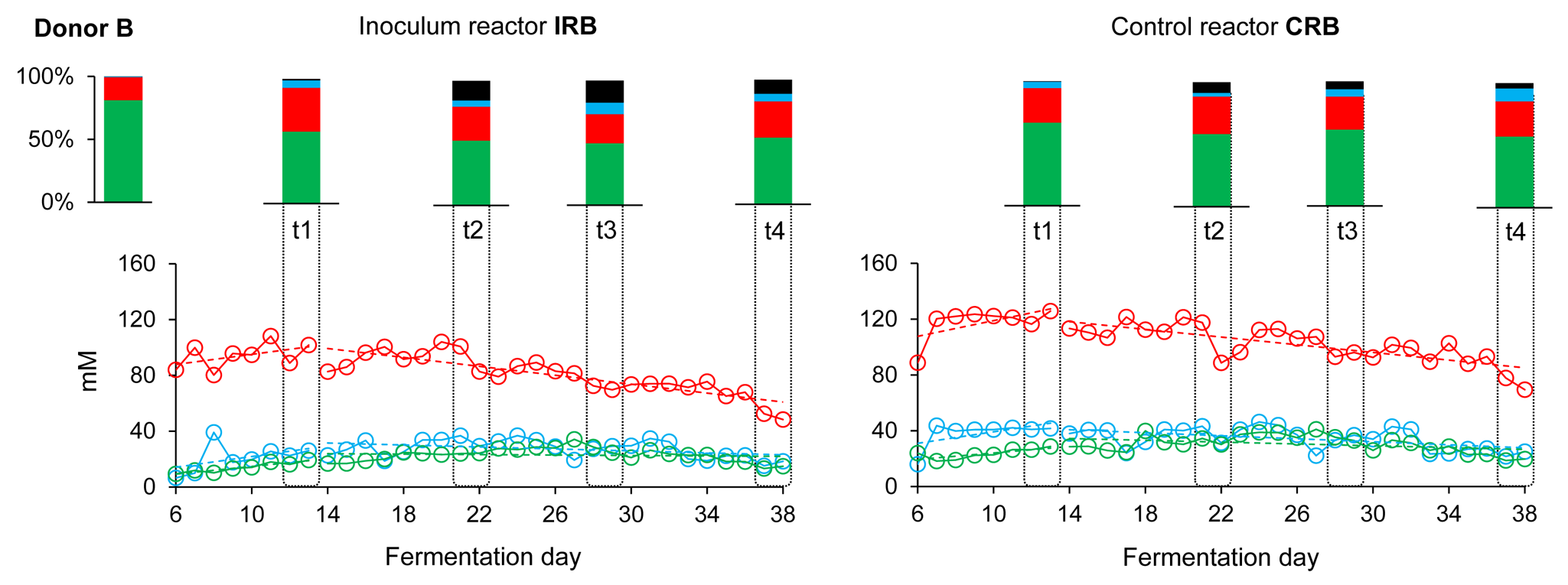

Supplement: Figure S2 — Microbiota composition and metabolic activity of the PolyFermS model B over time. Open circles visualize daily concentrations (mM) of acetate (in red), propionate (in blue) and butyrate (in green) in reactor effluents of IRB and CRB. Colored bars correspond to ratios (%) of Firmicutes (in green), Bacteroidetes (in red), Actinobacteria (in blue) and Proteobacteria (in black) detected in donor sample B and reactor effluents obtained at time points t1, t2, t3 and t4 from inoculum reactor IRB and control reactor CRB. (TIF) [file pone.0077772.s002.tif]

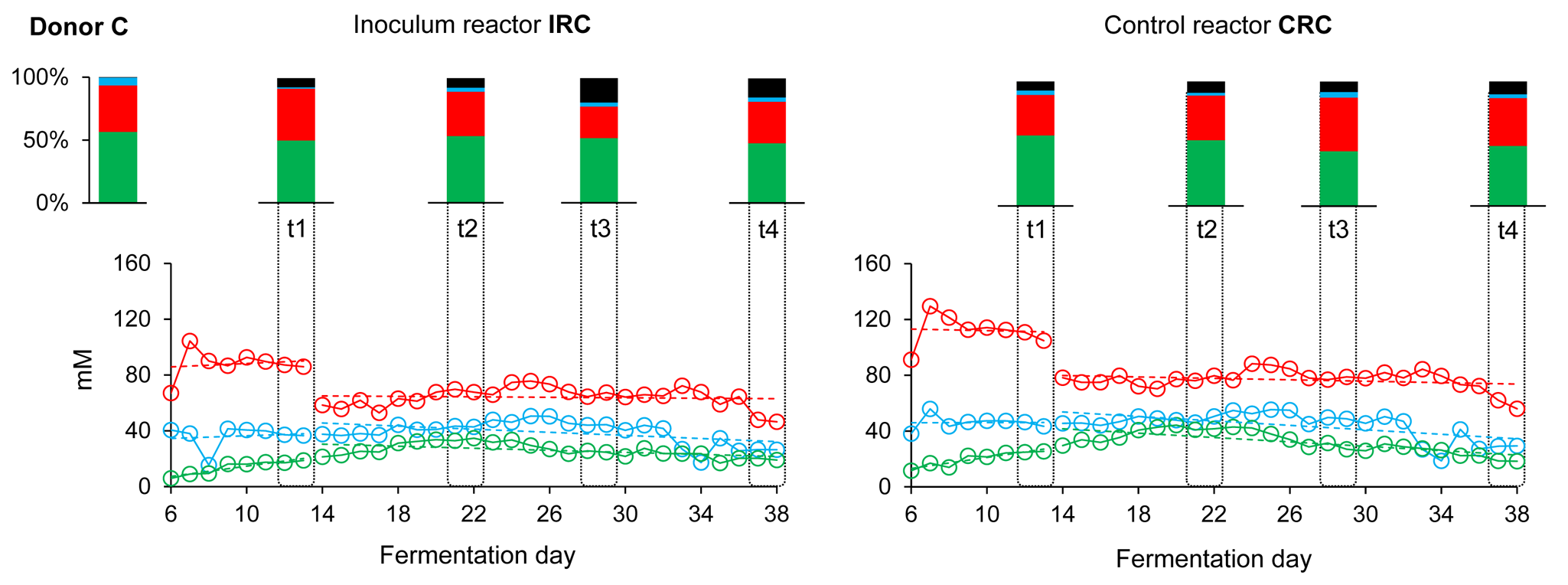

Supplement: Figure S3 — Microbiota composition and metabolic activity of the PolyFermS model C over time. Open circles visualize daily concentrations (mM) of acetate (in red), propionate (in blue) and butyrate (in green) in reactor effluents of IRC and CRC. Colored bars correspond to ratios (%) of Firmicutes (in green), Bacteroidetes (in red), Actinobacteria (in blue) and Proteobacteria (in black) detected in donor sample C and reactor effluents obtained at time points t1, t2, t3 and t4 from inoculum reactor IRC and control reactor CRC. (TIF) [file pone.0077772.s003.tif]
